# Involvement of the enteroendocrine system in intestinal obstruction

**DOI:** 10.1371/journal.pone.0186507

**Published:** 2017-11-01

**Authors:** Quentin Ballouhey, Laurence Richard, Laurent Fourcade, Ines Ben Rhaiem, Jean Michel Vallat, Franck Sturtz, Sylvie Bourthoumieu

**Affiliations:** 1 EA6309 peripheral neuropathy, University of Medecine, Limoges, France; 2 Department of pediatric surgery, University Hospital, Limoges, France; 3 Department of neurology, University Hospital, Limoges, France; 4 Department of biochemistry and molecular genetic, University Hospital, Limoges, France; 5 Department of histology, cytology and cytogenetic, University Hospital, Limoges, France; Laboratoire de Biologie du Développement de Villefranche-sur-Mer, FRANCE

## Abstract

**Introduction:**

Intestinal atresia, a rare congenital condition, is often associated with intestinal motility disorders despite adequate neonatal surgery. Previous studies have focused on changes in the enteric nervous system (ENS). We hypothesized that other components of the digestive tract could be involved in this condition.

**Material and methods:**

In a rat model of surgically-induced intestinal obstruction, a transcriptome analysis was performed to measure the global gene expression. Then, analyzes were focused on genes expressed in ENS and neuroendocrine cells. Rat fetus small intestines at different developmental stages (ED15, ED17, ED19 and ED21, (n = 22)) were studied as controls and compared to the upper and lower segments of small intestines from rat fetuses with surgically-induced obstruction (n = 14; ligature at ED18). The gene expression pattern was confirmed by immunohistochemistry, electron microscopy and RT-qPCR.

**Results:**

From ED15 to ED21, there was a physiological decrease in the gene expression of ENS markers and an increase in that of neuroendocrine genes. Regarding operated embryos, the changes in global gene expression were significantly higher in the proximal segment compared to the distal segment (18% vs. 9%). More precisely, a decrease in ENS gene expression and an increase in neuroendocrine gene expression were observed in the proximal segment compared to controls, indicating an accelerated maturation pattern. Immunohistochemistry and electron microscopy confirmed these findings.

**Conclusion:**

Fetal intestinal obstruction seems to induce an accelerated maturation in the proximal segment. Moreover, neuroendocrine cells undergo significant unexpected changes, suggesting that ENS changes could be associated with other changes to induce intestinal motility disorders.

## Introduction

Intestinal atresia is a common congenital gut disorder characterized by a disruption of intestinal continuity. The prevalence of small bowel atresia is of about 3 per 10,000 births [1–2] and its pathogenesis is unknown. No specific gene has been associated with isolated jejuno-ileal atresia [2]. Its treatment consists in surgical repair shortly after birth. The main postoperative complications affecting one third of all cases include intestinal dysmotility and bacterial translocations that may be life-threatening in severe cases.

The underlying pathophysiology of intestinal motility disorders is however not fully understood. Intestinal motility and secretions are initiated by luminal factors that activate intrinsic and extrinsic primary afferent nerves involved in peristaltic and secretory reflexes [3–4]. Postoperative intestinal motility disorders have been suggested to be secondary to enteric nervous system (ENS) changes, particularly in the region nearby intestinal atresia [5]. The histological assessment of specimens from patients with intestinal atresia has confirmed the presence of an impaired ENS structure in the proximal segment and a delayed maturation in the distal one [6]. A recent pathological study has reported atresia with extended proximal deterioration of myenteric ganglia in newborns [7]. These findings suggest that a potential intestinal segment should be resected at birth.

Various animal models of intestinal obstruction have been developed with a particular interest for chickens [8] and rats [9]. In the later model, pathological ENS patterns have been reported in both sides of obstruction. A histological study has identified an immature ENS pattern below the obstruction [10]. Additional investigations have shown very heterogeneous findings or morphological changes in both the proximal and distal segments [11].

The ENS is an integrative network consisting of neurons and glial cells derived from the neural crest. The precursor cells originate from neural crests and differentiate into various neuron subtypes [12] and mature glial cells [13]. Less than 20 neuron subtypes have been identified [3;14]. Enteric Neural Crest-derived Cells (ENCC) found in the small intestine originate from somites 1–7. The cells migrate according to a rostro-caudal gradient and give rise to the ENS. The entire length of the enteric tube is colonized at embryologic day 14 (ED14) in mice and 9.5 weeks of gestational age in humans. Neuron enteric subtype diversity [14] and electric activity [15] appear early in the development, before the end of the ENCC rostro-caudal migration. This rostro-caudal migration is followed by a secondary ENCC migration wave, which occurs in the radius of the developing gut [16]. During these rostro-caudal and centripetal migrations, ENCC differentiate into neuron subtypes and glial cells.

The ENS is located along the gut and regulates muscle contractions and secretions. It includes the myenteric plexus, which primarily controls peristalsis, and the submucosal plexus, which controls mucosal processes such as glandular secretion and mucosal blood flow [17]. Submucosal neurons are linked to the intestinal lumen via the neuroendocrine and glial cells. Neuroendocrine cells are distributed in the mucosa and, although they only account for 1% of epithelial cells, they form the largest endocrine system in humans [18]. They are exposed to the lumen of the intestinal tract acting as transducers and releasing hormones. New evidence suggests that they play a role in sensory nerve sensitization [19]. They could also play a significant role in the development of intestinal motility disorders.

The aim of this study was to exhaustively analyze the ENS and neuroendocrine cell development during gestation given their suspected involvement in intestinal dysmotility. For this purpose, a transcriptome analysis was performed to identify changes in gene expression during late normal intestinal development (between ED15 and ED21) and in a rat model of surgically-induced intestinal obstruction (ligature of the small intestine in rat embryos at ED18) [9]. The gene expression pattern was assessed by RT-qPCR, immunohistochemistry and electron microscopy.

## Materials and methods

### Ethics statement

The experimental protocol was approved by the French animal care and use committee (Comité National de Réflexion Ethique sur l’Expérimentation Animale; reference number 05180.04) and all animal procedures were performed according to the French guidelines for animal protection and welfare.

### Fetal bowels and intestinal obstruction

Pregnant Wistar rats were purchased from Janvier Labs. Pregnant rats were deeply anesthetized by inhalation of 3% isoflurane in combination with a mixture of nitrous oxide and oxygen (1:2, V/V). Kinetic control bowels at different developmental stages (ED15, ED17, ED19, ED21) were obtained after cesarean section and fetus laparotomy.

Intestinal obstruction was surgically induced in fetuses of pregnant Wistar rats, at day ED18 [9]. We selected well-exposed fetuses. The fetal abdominal wall was opened and an intestinal loop was extracted without causing liver or umbilical cord injury. A 10/0 Prolene (Ethicon, France) suture was performed around the exposed intestine. The abdominal loop was reintegrated into the abdominal cavity and then closed. During the same procedure, simple laparotomy was performed in other fetuses, which were used as sham controls. At day ED21 and to prevent cannibalism, all animals were delivered by cesarean section.

**Fig 1:**
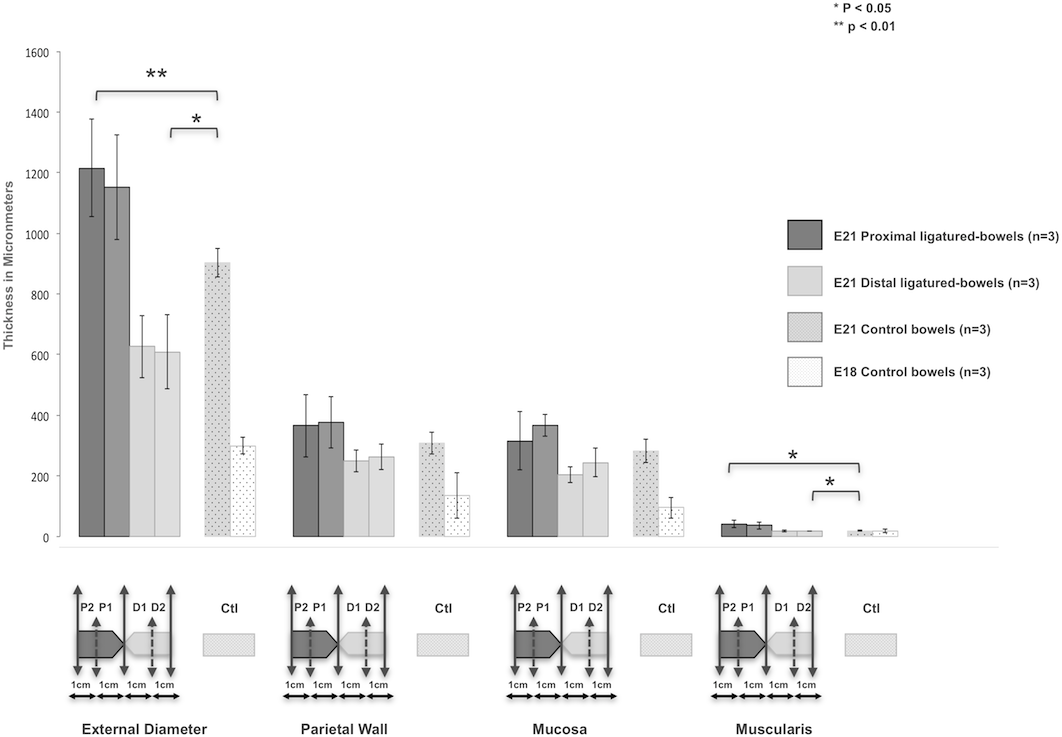
Morphometry of the intestinal wall. The outer diameter and muscle thickness were significantly (p <0.05) greater in the proximal segments than in the distal segments and in controls whereas the parietal wall and mucosa thickness were not affected by the ligature. No statistical difference was found between segments 1 and 2 of each part of the ligature in terms of diameter, wall, mucosa and muscularis layer measurements. Abbreviations: P1: proximal segment 1, P2: proximal segment 2, D1: distal segment 1 and D2: distal segment 2.

Studied fetuses were weighed and measured. Bowels were collected and measured. The criteria for successful surgery were: (i) a living fetus with a precise gestational age and (ii) macroscopic evidence of intestinal obstruction, i.e. a proximal intestine filled with biliary fluid and dilation of at least 2 cm distinct from a consistently uncolored distal segment. Obstructed bowels were analyzed on both sides of the ligature. For each experiment, two successive samples of 1 cm in length were taken in the proximal (P1 and P2) and distal (D1 and D2) segments as shown in Fig 1.

### RNA isolation

Total RNA were extracted from bowels using QIAzol® Reagent, and then treated with DNase I prior to purification using a Qiagen RNeasy Mini Kit according to the manufacturer's instructions (Qiagen, Hilden, Germany). Concentration and purity of eluted RNA were analyzed by spectrophotometry using the NanoDrop® ND-2000/2000c (Thermo, Waltham, Massachusetts, USA). RNA quality and integrity were assessed with the Agilent 2100 bioanalyzer using the RNA 6000 Nano Kit (Agilent Technologies, Santa Clara, CA). Only RNA samples with a RNA integrity number (RIN) greater than 7 were used. Samples were stored at −80°C until use.

### Microarray screening and data analysis

Affymetrix GeneChip® Rat Gene 2.0 Array was used to analyze differential gene expression profiles of control intestines at different stages of maturation (between ED15 and ED21, n = 16) and the segments proximal and distal to intestinal obstruction (ED21, n = 8). Samples were prepared according to the manufacturer’s instructions and recommendations (Affymetrix, Santa Clara, CA). Briefly, total RNA from each sample was reverse-transcribed, converted into complementary RNA using the standard Affymetrix protocol (Affymetrix, Santa Clara, CA, USA) and hybridized to the Affymetrix GeneChip Rat Gene 2.0 Array at the genomic platform of the Cochin Institute.

### Data processing and analysis

Raw Affymetrix data (.cel files) from arrays were transformed by the Robust Multiarray Analysis (RMA) method using Bioconductor in R software to obtain genome-level expression values. The probe-level raw intensities were background-corrected using RMA, and then quantile normalized and summarized into log-expression values.

### Statistical analysis

Normalized scanned probe array data were compared between groups to generate p-value and signal log ratio (fold changes). Changes in gene expression were analyzed using unsupervised hierarchical clustering (with log-transformed and normalized signal intensity values) and principal component analysis (PCA) to assess data from technical bias and outlier samples, and visualize intergroup differences. To identify differentially expressed genes, an analysis of variance (ANOVA) was used for each gene and pairwise Tukey’s post-hoc tests were used for comparisons between groups. Differences were considered significant when p was <0.05.

### Quantitative real-time PCR

Complementary DNA (cDNA) was obtained by reverse transcription (RT) of total RNA (0.5 μg) with QuantiTec Reverse Transcription Kit following the manufacturer's instructions (Qiagen, Hilden, Germany). Two RTs were performed for each RNA sample. All cDNAs were quantified using a Nanodrop ND-2000/2000c Spectrophotometer (Thermo, Waltham, Massachusetts, USA) to determine the concentration and purity of each sample. Primers for the genes of interest and the reference genes were designed using PrimerBlast (NCBI) and Primer3 software with melting temperatures ranging from 62°C to 63°C. Forward and reverse primer sequences used for real-time PCR amplification are shown in Table 1. Real-time PCR amplifications were performed using RotorGene SYBR Green PCR Kit (Qiagen, Hilden, Germany) in a total volume of 25 μL consisting of 1X RotorGene SYBR Green MasterMix, 600 nM of each primer and 80 ng of cDNA. The amplification was carried out using the Rotor-Gene 6000 multiplex system (Corbett Research) with the following qPCR conditions: 95°C for 10 min followed by 40 cycles of 95°C for 30 s and 62°C for 45 s. Each cDNA sample was run in duplicate for the genes of interest and in triplicate for the reference genes. Negative (water) controls were included in all qPCR runs. A PCR was also performed with each primer set in the presence or the absence of RT as controls.

**Table 1:** Primer sequences used for gene expression assessment by RT-qPCR.

Genes	Accession	Primer sequences 5’-3’
		Forward (F) / Reverse (R) primers
**Sst**	**NM_012659**	F: GACCCCAGACTCCGTCAGTTTC
		R: GGGTTCGAGTTGGCAGACCT
**Pyy**	**NM_001034080**	F: CGGCAGCGGTATGGGAAAAG
		R: ACACCTTCTGGCCGAGACCT
**Tph1**	**NM_001100634**	F: TGGGGGACCATCTTCCGAGA
		R: CGGATGGAAAACCCTGTGCG
**Calb2**	**NM_007586**	F: AGATGGGAAAATTGAGATGGCAGA
		R: GCTGCCTGAAGCACAAAAGGAA
**Chat**	**NM_009891**	F: TTAGCTACAAAACTCTGCTGGACA
		R: CACGATGACATGCTCAGGCTC
**Cck**	**NM_012829**	F: ATACATCCAGCAGGTCCGCAAA
		R: AGCCCATGTAGTCCCGGTCA
**Sct**	**NM_001287171**	F: CGGACGGGACGTTCACCAG
		R: CAGAAGCCAATCCTGTAGGGC
**Calb1**	**NM_031984**	F: ATATGGGCAGAGAGATGATGGGAA
		R: CCTCGCAGGACTTCAGTTGCT
**Gast**	**NM_012849**	F: GGTCCGCAACACTTCATAGCAGATC
		R: TACGGCGACCAAAGTCCATCC
**Ache**	**NM_172009**	F: CAGGTGCTGGTGGGTGTGGTG
		R: CCAGGTCACTCGCTTGGGGT
**Tbp**	**NM_001004198**	F: TGGGATTGTACCACAGCTCCA
		R: CTCATGATGACTGCAGCAAACC
**Hprt**	**S79292**	F: CTCATGGACTGATTATGGACAGGAC
		R: GCAGGTCAGCAAAGAACTTATAGCC

Abbreviations: Sst: Somatostatin, Pyy: Peptide YY, Tph1: Tryptophan hydroxylase 1, Calb2: Calbindin2, Chat: Cholineacetyltransferase, Cck: Cholecystokinin, Sct: Secretin, Calb1: Calbindin1, Gast: Gastrin, Ache: Acetylcholinesterase, Tbp: TATAbox binding protein, Hprt: Hypoxanthine-guanine phosphoribosyltransferase.

Data were analyzed using the corresponding software version 1.7 (Corbett Research). Gene expression was quantified using the relative delta Ct method by comparing the number of cycles of target genes with the internal reference genes (HPRT and TBP). The ratio of the target gene to the reference gene was calculated and expressed as 2-ΔCt. This ratio was then used to evaluate the expression level of the target gene in each sample.

Data are expressed as the mean of duplicates of three independent samples (mean ± SD) in three groups: proximal, distal and control intestines at ED21. Differences in gene expression were analyzed using a Student’s t-test. Differences were considered significant when p was <0.05.

### Optic microscopy

Bowels were collected, oriented and fixed immediately on ice in 4% paraformaldehyde (PFA) in PBS (phosphate-buffered saline solution, pH 7.4). After 3 hours, PFA was removed, and bowels were rinsed with PBS and incubated in 30% (w/v) sucrose in PBS overnight at 4°C. Samples were frozen by immersion in liquid nitrogen-cooled N-methyl butane. The proximal and distal segments on both sides of atresia were embedded together from the ligature. Eight-micrometer thick sections were cut using a freezing microtome and processed for immunohistochemistry or histology. For histology, sections were stained with hematoxylin and eosin for visualizing the overall morphology.

For immunohistochemistry, frozen cross sections were incubated in 10% normal serum in PBS for 1 h and then incubated overnight at 4°C with primary antibodies. The labeling was visualized using an appropriate avidin-biotin peroxidase kit (Vectastain; Vector Laboratories, Burlingame, CA) with 3,3-diaminobenzidine tetrahydrochloride (DAB) as the chromogen (DAB peroxidase substrate kit; Vector Laboratories). Sections were counterstained with Mayer’s hematoxylin, mounted, and observed with a Nikon optic microscope H600L (Nikon, Tokyo, Japan). The following primary antibodies were used: rabbit polyclonal anti-calretinin antibody (CALB2; Abcam ab 702, 1:100 dilution); rabbit polyclonal anti-chromogranin A antibody (CHGA, Abcam ab 45179; 1:200 dilution). The number of chromogranin-positive cells and calretinin-positive large ganglial spaced knots were quantified.

### Transmission electron microscopy (TEM)

Samples were fixed in 2.5% glutaraldehyde in 0.05 mol/L sodium cacodylate buffer, post-fixed in 1% osmium tetroxide, and embedded in epoxy resin. Semi-thin sections (1-μm thick) were stained with toluidine blue and examined under a light microscope. Areas appropriate for further ultrathin sectioning were then selected and examined using a transmission electron microscope. Ultrathin sections were stained with uranyl acetate and lead citrate and examined with an electron microscope JEOL JEM-1011 at 80KeV. Each obstructed bowel was compared to a control bowel in order to analyze tunica muscularis and unmyelinated fiber ultrastructures.

## Results

### Morphological analysis

A macroscopic analysis was performed on bowel sections of rat fetuses (ED21) with successful prenatal obstruction (n = 18) and controls (n = 12). Mean birth weight was 4.3 ± 0.5 g and mean total small bowel length was 11.5 ± 2.8 cm. Littermate mean birth weight was 5.1 ± 0.4 g and mean intestinal length was 16.8 ± 2.4 cm.

Atresia was located at a median distance of 6 cm (range 2–11 cm) from the duodeno-jejunal junction. Microscopy analysis carried out on the proximal and distal ligated gut sections (n = 3) and control gut sections (n = 3) showed that the outer diameter was significantly larger in segments proximal and distal to the ligature than in control segments (1.8 ± 0.1-fold; p <0.01 and 1.4 ± 0.1-fold; p <0.05, respectively). The muscle layer thickness was significantly (p <0.05) greater in the segments proximal to atresia than in the distal segments (1.9 ± 0.3-fold) and in controls (2.0 ± 0.3-fold) whereas the mucosa thickness remained unchanged after ligature (p >0.05). No statistical difference was found between segments 1 and 2 of each part of the ligature in terms of diameter, wall, mucosa and muscularis measurements (p >0.05). All the results are reported in Fig 1.

### Global gene expression analysis

This analysis was performed to determine at what embryological stage and in which intestinal segment gene expression was the most impaired. Microarray gene expression was studied in proximal and distal ligated (n = 4) and control bowel samples at different stages of differentiation: ED15 (n = 4), ED17 (n = 4), ED19 (n = 4) and ED21 (n = 4). During bowel differentiation, the number of genes significantly up- or downregulated was higher between ED17 and ED19 (Table 2). Compared to ED21 controls, 3,625 genes were significantly up- or downregulated in the segment proximal to the ligature (18% of the 20,267 genes) whereas the expression of only half of the genes (1894 genes, 9%) significantly changed in the distal segment (Table 2). The ligature thus appeared to have a stronger effect on the proximal segment.

**Table 2:** Whole transcriptome analysis of fetal rat small intestines.

	Controls E17 vs. E15	Controls	Controls		Proximal vs. E21 controls		Distal vs. E21 controls
		E19 vs. E17	E21 vs. E19				
Up- or down-regulated genes, p<0.05	3853	6671	6032		3625		1894
							
Upregulated genes, p<0.05	2046	2597	2441		1452		743
Downregulated genes, p<0.05	1807	4074	3591		2173		1151

The whole genome expression was studied in control fetal rat intestines and in obstructed intestine segments proximal and distal to atresia. The number of genes with a significantly changed expression (> or <1.2 folds) was calculated. For control rats, gene expression was compared at different developmental stages: ED15, ED17, ED19, ED21. No difference was found at ED21 between sham controls and kinetic controls regarding morphometry, gene expression and immunochemistry. Both control types of were thus called ED21 controls. For atretic rat fetuses, the ratio of up- or downregulated genes compared to ED21 controls was 18% (3625 genes) in the proximal segment while it was only 9% (1894 genes) in the distal segment.

### ENS and neuroendocrine gene expression analysis

The genes specifically expressed in the enteric nervous and endocrine systems were analyzed (S1 Table). In control rats, the gene expression of ENS markers was decreased between ED15 and ED21. In operated fetuses, an overall decrease in gene expression was observed compared to ED21 controls in the proximal segment but not in the distal segment. The differentiation process appeared to progress normally in the proximal segment. Regarding neuronal subtypes, few changes were observed in obstructed bowels, except for the acetylcholinesterase gene that was downregulated in both segments.

**Fig 2:**
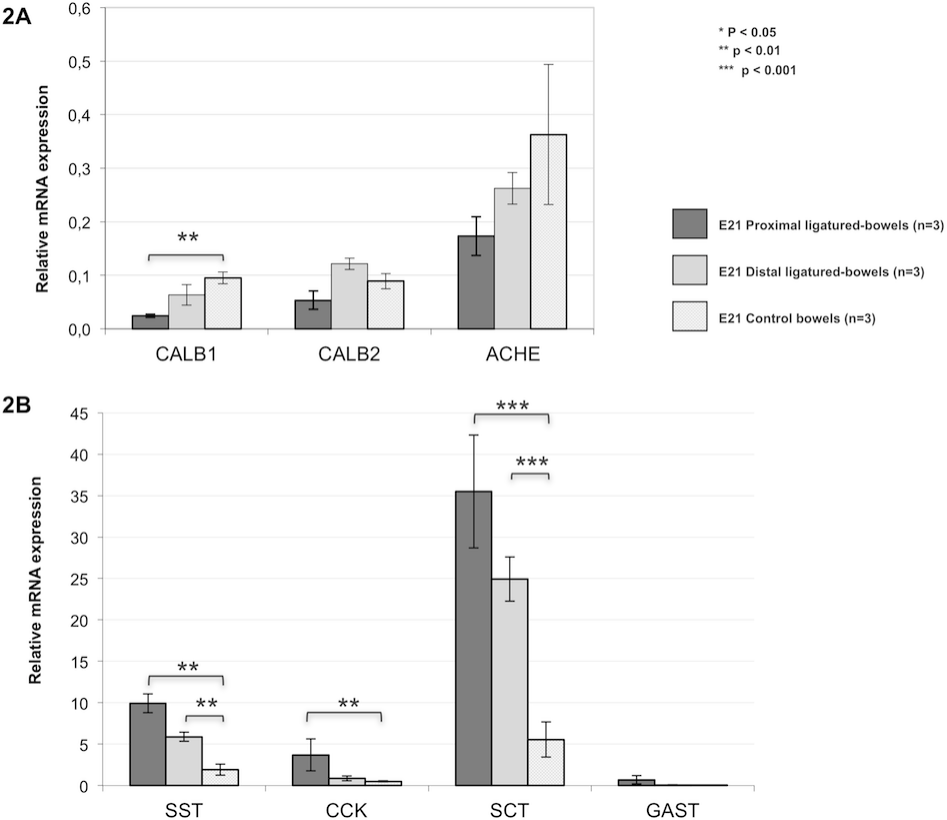
Assessment of gene expression by RT-qPCR in the enteric nervous and endocrine systems. (A) Enteric Nervous System gene expression: calbedin and calretinin were significantly decreased in the proximal segments compared to ED21 controls. (B) Endocrine cell gene expression: somatostatin, cholecystokinin, gastrin and secretin were significantly increased in the proximal segments compared to controls.

Conversely, the gene expression of hormones secreted by neuroendocrine cells increased during bowel maturation and was upregulated in the proximal bowel, except for peptide YY. The ligature did not seem to affect neuroendrocrine cell marker gene expression in the distal segment (S1 Table). These findings were confirmed by RT-qPCR (Fig 2) and IHC was performed to determine whether these changes in gene expression were also observed at the protein level.

### Immunohistochemistry

**Fig 3:**
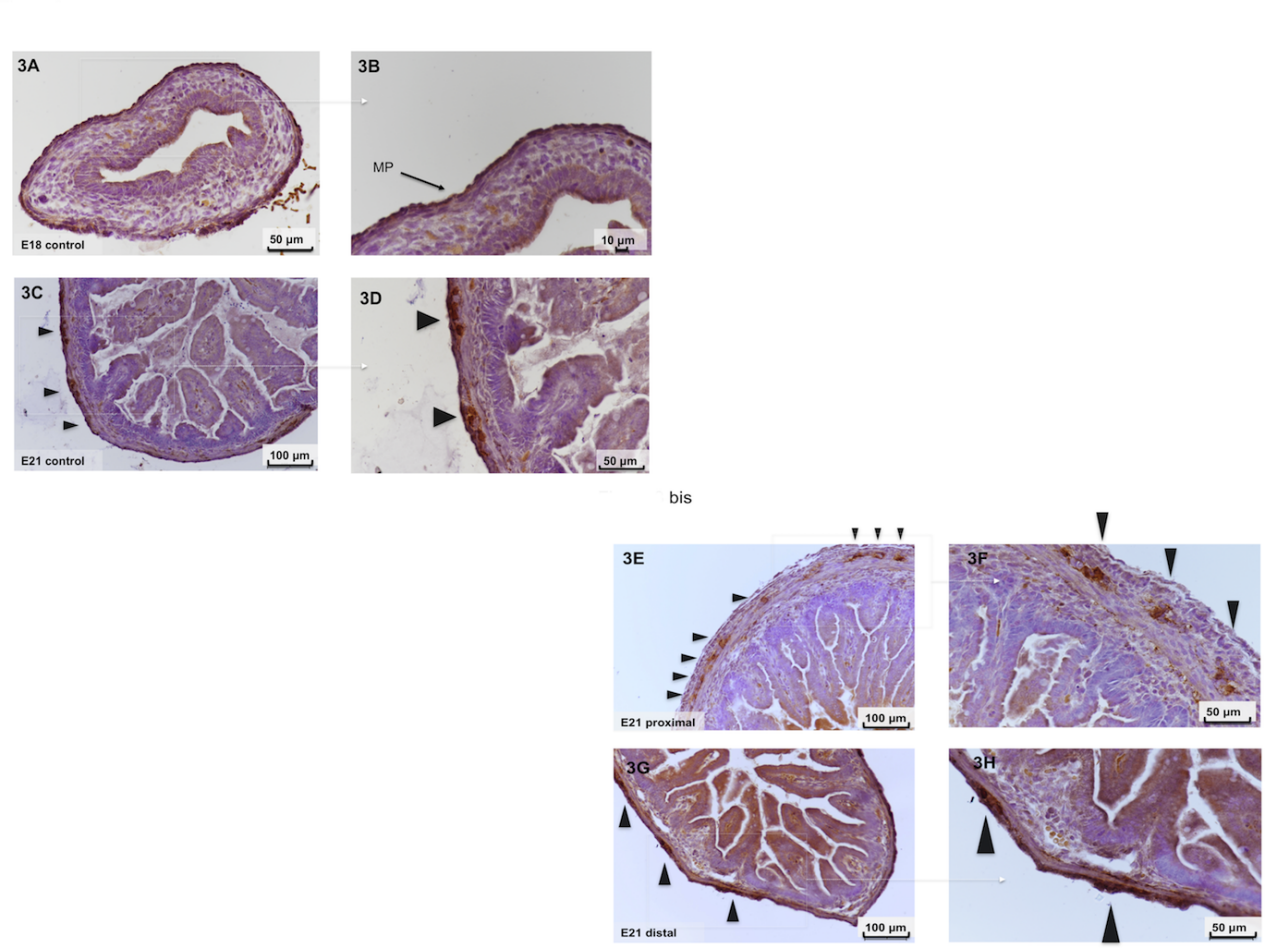
Immunohistochemistry of fetal intestinal sections. Immunohistochemistry performed using an anti-calretinin antibody in ED18 controls (A and B), ED21 controls (C and D), proximal segments (E and F) and distal segments (G and H) (magnification X20 and X40, respectively). Black arrows indicate the myenteric plexus located between the circular and longitudinal layers of muscularis tunica. (F) calretinin staining showing the presence of large and round myenteric ganglia in the proximal segments whereas ganglia were more elongated in the distal segments (H) and in ED21 controls (D).

**Fig 4:**
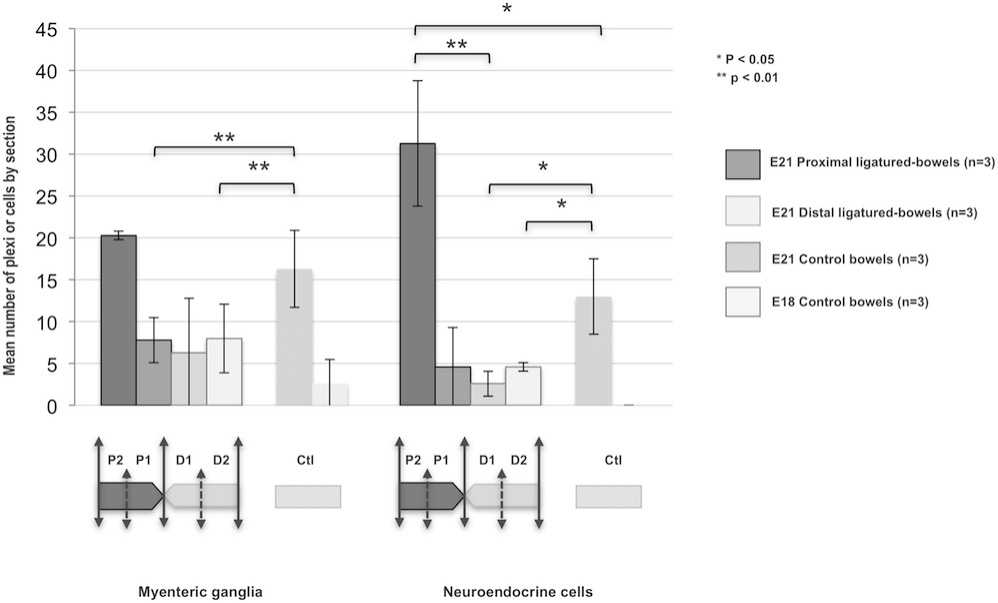
Quantification of the myenteric plexus and neuroendocrine cells in fetal intestinal sections. Calretinin staining showed a significant increase in the number of myenteric ganglia in the proximal segments compared to ED21 controls and distal segments (p <0.05). Chromogranin A staining showed a significant increase in the number of neuroendocrine cells in the proximal segments compared to the distal ones (p <0.05). This increased staining involved proximal segments 2 (P2) but not proximal segments 1 (P1) (p <0.05). A significant decrease was also observed in the distal segments compared to controls.

Myenteric ganglia were easily quantified with calretinin staining between the two layers of tunica muscularis (Fig 3). In controls, at ED18, a continuous ring of cells was observed (Fig 3A and 3B), and at ED21 this structure was clearly separated by nodes (Fig 3C and 3D). The staining pattern of the distal segment was similar to that observed in ED21 controls (Fig 3G and 3H). Regarding the proximal segment (Fig 3E and 3F), large myenteric ganglia spaced knots were observed. The number of calretinin-positive myenteric ganglia was significantly increased in the proximal segment compared to controls (p <0.01) (Figs 3 and 4). This increased staining was only observed in the proximal segment P2, but not in the P1 segment (p <0.01). No significant difference was found between the distal segments D1 and D2 (Fig 4) and controls.

**Fig 5:**
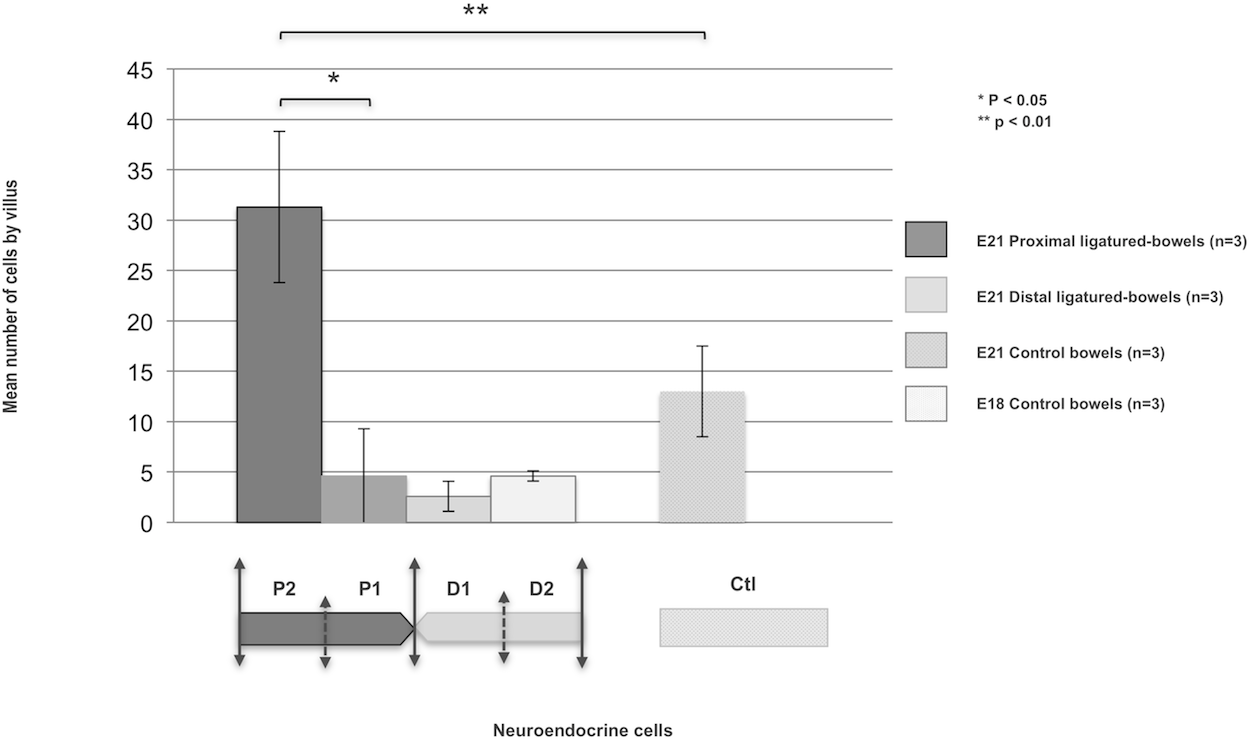
Quantification of neuroendocrine cells per villus in fetal intestinal sections. Chromogranin A staining showed a significant increase in the number of neuroendocrine cells in the proximal segments compared to controls (p <0.05). This increased staining involved proximal segments 2 (P2) but not proximal segments 1 (P1) (p <0.01).

Chromogranin A staining showed a significantly increased number of neuroendocrine cells in the proximal segment compared to the distal segment (p <0.05) (Figs 4 and 5). This increased staining was only observed in the P2 segment but not in the P1 segment (p <0.01). Chromogranin A staining was also significantly decreased in the D1 and D2 segments compared to controls (p <0.05) (Fig 4). No chromogranin A staining was detected at ED18.

### Electron microscopy

**Fig 6:**
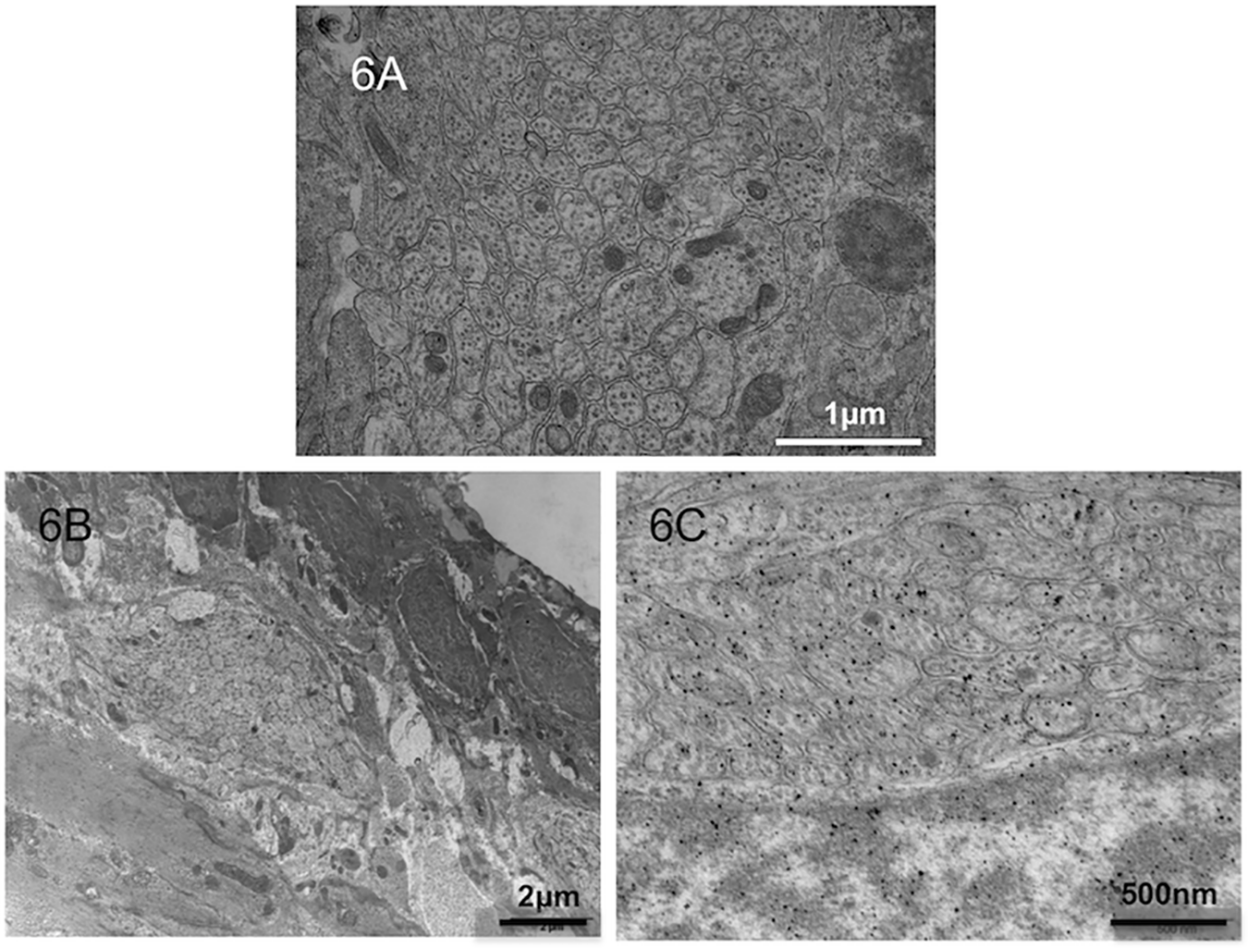
Electron microscopy of fetal intestinal sections. Electron microscopy focusing on unmyelinated fibers at ED21 in controls (A) and in the proximal segments (B). In the distal segments (C), a reduced number of unmyelinated fibers was observed.

In controls, transmission electron microscopy showed that the myenteric ganglia and unmyelinated fibers were located between the circular and longitudinal muscle layers (Auerbach’s plexus). Mitochondria were present within unmyelinated fibers (Fig 6A).

In the proximal segments, the number of unmyelinated fibers was reduced and the number of nerve cells per ganglion was higher than normal (Fig 6B). In the distal segments, the number of unmyelinated fibers was also reduced (Fig 6C).

## Discussion

To our knowledge, this is the first study exploring both intestinal obstruction and gestational development using a global transcriptomic approach with a special focus on the ENS and neuroendocrine system. Surprisingly, we observed a downregulation of the genes encoding panneuronal markers during gut differentiation (from ED15 to ED21) whereas the gene expression was upregulated in neuroendocrine cells. Regarding the global gene expression, the effect of the ligature was stronger in the proximal segment than in the distal one. In distal segments, the gene expression patterns and gut morphology were fairly similar to those in controls. In contrast, in proximal segments, significant morphological changes were observed with gut dilation, muscle hypertrophy and neuroendocrine cell hyperplasia. In this proximal segment, the changes in gene expression were similar to those observed during differentiation, i.e. downregulation of all neuronal markers and upregulation of neuroendocrine cell genes. Together, these kinetic patterns suggest an accelerated maturation of the obstructed proximal bowel segment.

Morphologically, our results confirmed the global changes previously described in several animal models [9, 11, 20] with a large distension of the lumen and muscle hypertrophy above the obstruction. However, the debate is still ongoing in the literature between those promoting the impairment of the proximal segment [21–22] and those promoting that of the distal segment [6, 10] for explaining the postnatal pathophysiology of this condition. The morphological changes previously reported in animal models (S2 Table) and in human atresia (S3 Table) seem to affect more significantly the proximal segment than the distal one. Some publications have reported a similar expression of various parameters between the distal segment and controls [7, 8, 22–31]. Recent human studies support an involvement of the proximal segment, with significant abnormalities affecting the ENS and muscle layers, to explain postnatal intestinal motility disorders [7]. All these changes are actively debated but no study had so far used a systematic approach as we did.

According to our findings, the proximal segment appears to be more affected by the ligature given the significantly higher number of genes that were differentially expressed from the global transcriptome. The gene expression profile confirms the accelerated maturation of this segment [6]. This assumption is supported by the mechanical theory in the development process [32]. This theory supports the evidence that mechanical stresses induce mecanotransduction pathways with subsequent cell proliferation and tissue differentiation. The luminal fluid pressure changes and the interruption of the amniotic fluid flow induced by the ligature could impact the development of the proximal segment but not that of the distal segment.

### Enteric nervous system

Regarding the ENS development in the proximal segment, gene expression analysis and immunohistochemistry showed an accelerated maturation and an increased number of large myenteric ganglial spaced knots. These results confirm previous data with a morphological pattern of delayed maturation in the distal segment in a rat model [10], an accelerated maturation in the proximal segment [6] and an increased number of myenteric ganglia [22]. In the literature, morphological changes involve more significantly the proximal than the distal segment (S2 and S3 Tables) whereas the submucosal plexus appears to be more affected than the myenteric one [20, 24]. This could be due to the time at which ligature was performed in our model, i.e. after the end of the ENCC rostro-caudal migration (ED14 in rats) and before the end of the centripetal migration (ED17-ED19 in rats), leading to the formation of both plexuses [33–34].

Most genes encoding neurotransmitters are not affected by the intestinal ligature with the exception of the acetylcholinesterase gene which is the most expressed gene in the ENS [3]. This downregulation has previously been described by IHC in the proximal segment [29] although a higher proportion of ChaT neurons was found based on the mRNA expression [11]. Our results indicate a downregulation of acetylcholinesterase in both segments. They are in line with previously reported results (S2 and S3 Tables).

The present animal model [9] allows exploring the consequences of intestinal obstruction with a supposed late ligature time compared to congenital human atresia, due to technical limitations (fetus size). The prenatal natural history of intestinal obstruction in human atresia is still debated with an assumed occurrence between the second [8] and third trimester [35] of gestation. The onset is important because the regenerative potential of the ENS could be related to its degree of immaturity [22]. According to our model, the reduced number of differentially expressed genes involved in neuron and glial cell development confirms the previous assumption of a limited involvement of the ENS in motility disorders. Conversely, the neuroendocrine system seems to be more affected by the ligature.

### Neuroendocrine cells

The transcriptomic approach used in this study showed an increased number of differentially expressed genes encoding neuroendocrine markers. An increase in gene expression tended to be observed in control rat fetuses in late gestation, suggesting a particular role in the subsequent digestion process. In operated fetuses, a major increase in gene expression was found in the proximal segment compared to controls, whereas a reduced number of neuroendocrine cells was observed in the distal segment, suggesting a pathophysiological role in the impaired portion of the intestine. These findings have been previously reported in another animal model [25]. These changes also confirm an accelerated maturation in the proximal segment and a delayed maturation in the distal segment. This reorganization of neuroendocrine cells could also be due to the difference in pressure induced by the amniotic fluid on each side of the ligature. The enteroneuroendocrine system plays a key role in controlling gastrointestinal secretion and motility. In the small bowel, these specialized cells release various hormones: gastrin that stimulates enzyme secretion, somatostatin that inhibits gastrin secretion and motility, cholecystokinin that inhibits gastric motility and regulates glucose homeostasis, polypeptide YY that regulates food intake and 5-hydroxytryptamin that promotes intestinal motility. Through the chemosensory machinery, they are capable of sensing the luminal content and initiating gut-brain interactions [19]. This transmission to the ENS involves indirect interactions through neurohumoral peptide release [36] or direct cellular and neuronal connections through a basal process recently referred to as “neuropod” [37]. Disturbances of this critical communication could alter homeostatic gut functions. The recent literature has suggested a role of enteroendocrine cells in various gastrointestinal disorders, including inflammatory or autoimmune diseases potentially associated with subsequent dysmotility and malabsorption [38–39]. This interaction between the ENS and the endocrine system is also crucial for the intestinal functional maturation and emerging evidence strongly suggests that the microbiota could participate in its development [40]. Based on these results, postoperative motility disorders could be more closely related to the neuroendocrine system than to the ENS.

### Medical implications

The obstruction model shows a significant difference between the segments that are close to or remote from atresia. It suggests that a limited portion of the segment is particularly involved in both ENS and neuroendocrine changes with a particular attention to proximal segments.

The same trend has been reported by Ozguner et al [28] and Wang et al [7] who have found a pathological expression of neuronal markers particularly in the proximal segment. They have found disturbances up to 4 cm and 15 cm remote from atresia on pathological specimens and then suggested to resect the equivalent length in the proximal bowel to prevent neonatal disorders [7]. Our results confirm the predominant abnormal presentation of the proximal segment and suggest that when surgical resection is discussed, it should include the proximal segment.

Our results show a significant reorganization of the neuroendocrine system that could play a role in dysmotility secondary to intestinal obstruction. Treatments targeting these hormonal pathways could be considered as an adjunct to surgery to prevent postoperative complications.

## Conclusion

Intestinal obstruction impairs gut development, mainly in the proximal segment, and accelerates maturation. The distal segment of the obstructed intestine is only slightly affected by these changes. The proximal segment probably undergoes pathological intraluminal pressure due to the interrupted flow of amniotic fluid. Our global approach in the obstruction model suggests that the involvement of the ENS is limited in this pathological gut development. In contrast, neuroendocrine cells underwent significant unexpected changes related to obstruction, supporting the evidence that the ENS does not play an exclusive role in intestinal motility disorders. Further research is needed to determine the real impact of the neuroendocrine system on neonatal disorders before assessing it as a therapeutic target.

## Supporting information

S1 Table Transcriptome analysis focused on ENS and neuroendocrine cell gene expression. Fold changes of the gene expression and p-values **were** measured for control fetuses at ED21 vs. ED15 (n = 4). Similarly, the proximal and distal segments (Prox and Dist, respectively) of operated fetuses were compared to ED21 controls (n = 4 in each group). Overall, ENS gene expression was physiologically decreased from ED15 to ED21 in controls. Some decreases were also observed in the proximal segment of atretic fetuses but no change was observed in the distal segment. For neuroendocrine cells, the expression of several genes was increased during bowel maturation in control fetuses. In operated fetuses, many genes were upregulated in the proximal segment, except for peptide YY, while the distal segment appeared much less affected by the ligature.
(XLSX)

S2 Table Literature review of enteric nervous system and muscle changes in animal models of intestinal atresia. Abbreviations: ED: embryonic development, ICC: interstitial Cell of Cajal (pacemarker cells), NADPH-d: NADPH-diaphorase, α-SMA: alpha-smooth muscle actin, ChAT: choline acetyltransferase, AChE: acetylcholinesterase, CALB2: calretinin, NSE: neuronal specific enolase, NF: neurofilament, GFAP: glial fibrillary acidic protein, nNOS: neuronal nitric oxide synthase, SIS: staining index for synaptophysin, VIP: vasoactive intestinal polypeptide, SP: substance P, TEM: transmission electron microscopy, SEM: scanning electron microscopy, IHC: immunohistochemistry, HE: hematoxylin and eosin staining, HES: hematoxylin, eosin, Saffron staining, IR: immunoreactivity.
(XLSX)

S3 Table Literature review of muscle and nervous changes in series of patients with intestinal atresia. Abbreviations: GA: gestational age, ICC: interstitial Cell of Cajal (pacemarker cells), NADPH-d: NADPH-diaphorase, α-SMA: alpha-smooth muscle actin, ChAT: choline acetyltransferase, AChE: acetylcholinesterase, CALB2: calretinin, NSE: neuronal specific enolase, NF: neurofilament, GFAP: glial fibrillary acidic protein, nNOS: neuronal nitric oxide synthase, SIS: staining index for synaptophysin, VIP: vasoactive intestinal polypeptide, SP: substance P, TEM: transmission electron microscopy, SEM: scanning electron microscopy, IHC: immunohistochemistry, HE: hematoxylin and eosin staining, HES: hematoxylin, eosin, Saffron staining, IR: immunoreactivity.
(XLSX)
